# Effectiveness of influenza vaccination of schoolchildren in the city of São Paulo, Brazil, 2009

**DOI:** 10.1111/irv.12328

**Published:** 2015-10-13

**Authors:** Vera L Gattás, Maria Regina A Cardoso, Gabriela Mondini, Clarisse M Machado, Expedito J A Luna

**Affiliations:** a Butantan InstituteSão Paulo, Brazil; b School of Public Health, University of São PauloSão Paulo, Brazil; c Institute of Tropical Medicine, University of São PauloSão Paulo, Brazil

**Keywords:** Effectiveness, influenza, intervention study, school-age children, vaccination

## Abstract

**Background:**

Children play an important role in maintaining the transmission of influenza. Evidence suggests that vaccination of school-age children can reduce transmission to unvaccinated household contacts. We evaluated the direct and indirect effectiveness of the 2009 inactivated seasonal influenza vaccine in vaccinated schoolchildren and their unvaccinated household contacts.

**Methods:**

This was a double-blind cluster randomized trial involving 10 schools and 1742 schoolchildren as well as 5406 household contacts. The schools were randomly assigned to receive the influenza vaccine or the control vaccine. After vaccination, the schoolchildren and household contacts were followed for 6 months to identify cases of acute respiratory infection (ARI). Reverse-transcriptase polymerase chain reaction was performed for the diagnosis of influenza.

**Results:**

A total of 632 ARI cases were detected. Of those, 103 tested positive for influenza virus (influenza virus A[H1N1]pdm09 virus in 55 and seasonal influenza viruses in 48). The effectiveness of the vaccine in protecting against seasonal influenza virus infection was 65·0% for the household contacts (95% CI, 19·6–84·3) and 65·0% for the schoolchildren (95% CI, 20·9–84·5).

**Conclusion:**

Vaccination of schoolchildren significantly protected them and their household contacts against seasonal influenza.

## Introduction

Influenza, or flu, is a globally distributed, highly transmissible acute viral infection of the respiratory system.^1^ Because they are highly transmissible and mutable, influenza viruses, principally influenza A viruses, commonly cause annual outbreaks, epidemics, and sometimes pandemics resulting in high morbidity and mortality.^2^

Since the 1957 influenza pandemic, it has been observed that children play a central role in disseminating the influenza viruses.^3^ Studies have also shown that, because school-age children act as ‘reservoirs’ and ‘vectors’ of the influenza viruses, they are major disseminators of influenza in the community.^4^,^5^

Influenza has effects that extend beyond the disease itself, increasing school absenteeism and work absenteeism (as a result of parents or legal guardians having to stay home in order to look after their children). In addition, during the annual flu seasons, cases among schoolchildren increase the risk of another household member becoming ill within 3 days after one school day missed by the child.^6^ Influenza vaccination has been shown to confer protection against respiratory tract infections in household contacts of vaccinated children, as well as to reduce the rates of work absenteeism among parents and the number of medical visits for respiratory diseases.^7^,^8^ According to Glezen^9^, school-based influenza vaccine clinics are a reasonable option to improve vaccine coverage.

Reichert *et al*. 10 examined the indirect effect of the Japanese influenza vaccination program in the 1977–1987 periods, during which vaccination was compulsory for children in the 5- to 15-year age bracket. It is estimated that, on an annual basis, the program prevented 39 000–47 000 deaths from all causes, or in other words, one death prevented for every 420 vaccinated children. When the program was discontinued, the rates of excess mortality attributable to influenza among the elderly returned to previous levels. Therefore, mass vaccination of school-age children proved effective in inducing herd immunity in other sections of the population by breaking the chain of household transmission of the disease.

In the United States, mass vaccination with live attenuated influenza vaccine in the state of Maryland was found to have induced indirect immunity and, consequently, a reduction in the number of medical visits for acute respiratory infection (ARI).^11^

However, Jefferson *et al*. (2012) and Osterholm *et al*. (2012) conducted large meta-analyses, and considering the limited results on the effectiveness of vaccinating children, they recommended that more studies should be performed. 12,13

Since 1999, influenza vaccination campaigns have been conducted annually in Brazil, targeting the population over 60 years of age, health professionals, and patients with some chronic diseases. Instituto Butantan, one of the Brazilian public vaccine manufacturers, has begun to produce the influenza vaccine on a large scale. This increases the possibility of implementing new influenza vaccination strategies aimed at different population groups, such as school-age children. To date, no studies evaluating the effectiveness of influenza vaccination of schoolchildren in tropical countries located in Latin America were performed.

The primary objective of this study was to evaluate the direct effectiveness of influenza vaccination in preventing the disease in vaccinated schoolchildren and the indirect effectiveness of influenza vaccination in preventing the disease among their unvaccinated household contacts.

This study was planned in 2008. Vaccination of the study participants was scheduled to occur simultaneously to the National Influenza Campaign in the last week of April/first week of May. It actually occurred from 18 May 2009 to 22 May 2009. In the second week of April, Mexican health authorities reported the first cases of what would become the 2009 influenza pandemic. In April 24, the World Health Organization (WHO) issued the first pandemic alert. The first A(H1N1)pdm09 cases were reported in Brazil in the first week of May. The emergence of the pandemic meant that the dominant circulating influenza virus strain was not included in the vaccine whose effectiveness was being analyzed.

## Methods

This double blind, randomized community trial addressed the effectiveness of the 2009 Southern Hemisphere seasonal influenza vaccine in children and adolescents of 6–19 years in 10 schools, in selected school from the São Paulo State Department of Education Central-Western District of Education schools, and its indirect effectiveness among unvaccinated household contacts. The experimental group received the 2009 Southern Hemisphere inactivated seasonal influenza vaccine, and the control group received the meningococcal C conjugate vaccine. Controls under 9 years of age also received one dose of varicella vaccine. This was made because those under 9 years of age in the experimental group received two doses of the influenza vaccine, as recommended by the Brazilian Ministry of Health. None of the vaccines used in the trial were available in the Brazilian public vaccine program.

The inclusion criteria were as follows: being over 6 years of age, living in the study area, being enrolled in one of the selected schools, and having parents or legal guardians who understood the study procedures and gave written informed consent. The exclusion criteria were as follows: having a history of anaphylaxis or hypersensitivity to eggs, chicken protein, or other components of the influenza vaccine; having had a systemic hypersensitivity reaction to any drug or substance including neomycin, formaldehyde, the Triton X-100 (octoxynol 9), egg or chicken protein, or after the administration of this vaccine or any vaccine containing the same components; having severe acute illness (e.g. fever above 38·5°C and diarrhea, according to Gidudu 14) at the time of vaccination; being under treatment with immune suppressant drugs; and having received any other vaccine in the last 3 weeks.

To standardize the identified cases, we adopted the following definitions:

1. Acute respiratory illness (ARI) – a case of an individual with at least two of the following signs and symptoms: cough, rhinorrhea, sore throat, tachypnea, myalgia, headache, loss of appetite, and prostration. We have adapted the Brazilian surveillance definition and excluded fever, to make our definition more sensitive.^15^
2. Laboratory-confirmed influenza virus infection (LCI) – a case of ARI with influenza virus infection being confirmed by reverse-transcriptase–polymerase chain reaction (RT-PCR).
3. Influenza A(H1N1)pdm09 virus infection – a case of ARI with influenza virus infection confirmed by RT-PCR and the virus being positively identified as influenza A(H1N1)pdm09 virus.
4. Seasonal influenza virus infection – a case of ARI with influenza virus infection confirmed by RT-PCR and the virus not being positively identified as influenza A(H1N1)pdm09 virus.
5. Household contact – any individual who, during the study period, resided in the same household as did a schoolchild participating in this study.

The study protocol was approved by the ethical review board of the University of São Paulo – School of Medicine's Hospital das Clínicas (Protocol no. 1053/08), located in the city of São Paulo, Brazil.

### Adverse reactions

All vaccinated children were evaluated within the first 30 min after vaccination. Parents or legal guardians were asked to write down (on a spreadsheet designed specifically for this purpose) any vaccination-related adverse events occurring within up to 21 days after vaccination. In addition, during this period, the occurrence of adverse events was asked in the telephone calls. Thereafter (throughout the monitoring period), passive surveillance was used to detect adverse reactions.

### Sample size

Considering that no estimates on the incidence of laboratory-confirmed influenza were available for the Brazilian population, we decided to use a conservative estimate and assumed that about 2–4% of all children and adolescents would be laboratory-confirmed cases of influenza virus infection at some point during the influenza season.* With a statistical power of 80% and a significance level of 5%, we calculated that a sample size of 4588 children and adolescents in the 6- to 19-year age bracket (2294 allocated to the experimental group and the same number to the control group) would be enough to detect an indirect effect of vaccination as low as 20%. To select the sample, we used a systematic sampling procedure. Ten schools were randomly selected among the 29 schools of São Paulo State Department of Education Central-Western District of Education. Five schools were randomly allocated to the experimental group and the remaining five to the control group. All children enrolled in these schools, and their household contacts were invited to participate. Regarding household contacts, we assumed an average of four residents per household. Therefore, we would have approximately 13 764 household contacts (6882 being allocated to the experimental group and 6882 being allocated to the control group).

### Randomization and blinding

A statistician who was blinded to the study randomly allocated the schools to the intervention group (the influenza vaccine group) or the control group. The nurse who was responsible for the fieldwork was the only person who had access to information regarding which vaccine was used in each school. She did not participate in any other activity related to the study. The research assistants responsible for the active surveillance and home visits and the laboratory staff were also blinded.

### Vaccination

The schools were randomly selected to receive the 2009 Southern Hemisphere inactivated seasonal influenza vaccine 16 (Sanofi Pasteur SA, Lyon, France, in partnership with the Butantan Institute, Sao Paulo, Brazil – lot:0904070) or the meningococcal C conjugate vaccine (lot:YA1054A; Chiron Corporation, Emeryville, CA, USA). Children under 9 years of age in the influenza vaccine group received a second dose of the influenza vaccine 30 days after having received the first. Controls under 9 years of age received one dose of varicella vaccine (lot:2770210; Green Cross Corp., Yongin, South Korea) 30 days after having received the meningococcal C conjugate vaccine, to mimic the influenza vaccination schedule. All vaccines were administered in the deltoid muscle.

The head of the households where the children lived gave written informed consent to the participation of the children and the household members. Verbal assent was obtained from the participating children. In the 10 selected schools, vaccination occurred between 18 May 2009 and 22 May 2009.

### Active surveillance

Active surveillance began after vaccination and was conducted from 1 June 2009 to 30 November 2009. Active surveillance was conducted by telephone. The telephone calls followed the sequential enrollment numbers in the study and when the call was not completed, that number was automatically allocated to the next day's list. Approximately 30% of the sample was screened daily for ARI, so that every week all participating households were contacted at least once. Whenever a case of ARI was detected by active surveillance in any of the household members, a home visit was made to gather information on the case and collect a nasal secretion sample for diagnostic confirmation. The home visits were made in the same day the case was detected or in the following day. Nasal lavage technique was used for collection of nasal secretions. ARI cases occurring within 2 weeks after vaccination were not included in the analysis.

### Outcomes

The primary outcome measure was laboratory-confirmed influenza (LCI). Secondary outcomes were as follows: ARI, seasonal and pandemic influenza.

### Laboratory methods

Viral RNA extraction was performed with commercial nucleic acid extraction kits, including the QIAamp Viral RNA Mini Kit (QIAGEN, Hilden, Germany) and the GE Healthcare Viral RNA extraction kit (GE Healthcare, Chalfont, UK). After viral RNA extraction, reverse transcription of RNA to c-DNA was performed with the High Capacity c-DNA Reverse Transcription Kit (Applied Biosystems, Foster City, CA, USA). The real-time RT-PCR protocol for detection of influenza A(H1N1)pdm09 virus was in accordance with the recommendations of the United States Centers for Disease Control and Prevention.^17^

### Statistical analysis

After having characterized the schoolchildren by randomization group, we performed a descriptive analysis based on the incidence density of the outcomes studied by socio-demographic variables and household-related variables. The frequency of the study's outcomes was compared between the intervention group and the control group and between their respective household contacts. A Poisson regression model was used to estimate the risk ratios and respective 95% confidence intervals (CIs). The covariates included in the model were as follows: age group, sex, race, number of people sharing bedroom, and number of people in the household. The protective effectiveness of influenza vaccination was calculated by the following formula: ([1 − RR]*100). For the analysis of the effectiveness, it was considered the design effect, to correct the loss in precision due to the cluster sampling. Data analysis was performed with the stata software, version 11.1 (StataCorp LP, College Station, TX, USA).

## Results

**Figure 1:**
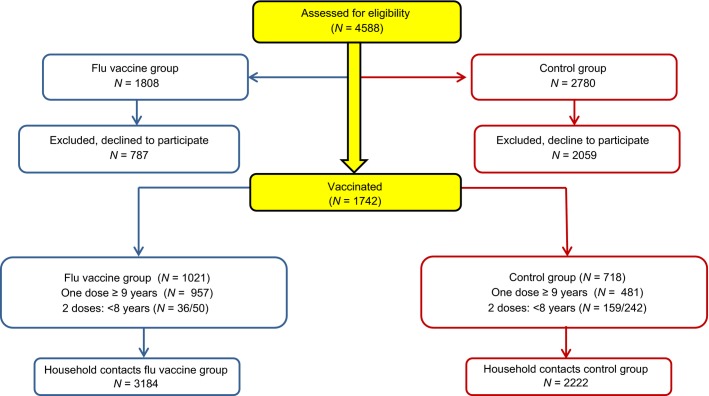
Flowchart of the community trial, Sao Paulo, Brazil, 2009.

Figure1 shows a flowchart of the community trial. Initially, 10 schools were randomly selected from among the 29 elementary schools in the study region. All of the schoolchildren enrolled in the selected schools were invited to participate in the study. Of those 10 schools, five (comprising 1808 enrolled students) were selected for the influenza vaccine group and the remaining five (comprising 2780 enrolled students) were allocated to the control group. The actual number of children enrolled in the schools allocated to the experimental group was smaller than expected from the school district data. The parents or legal guardians of 1742 (33·5%) of those children gave written informed consent. Of the 1742 children, 1021 (58·61%) were in the schools allocated to the influenza vaccine group and 721 (41·39%) were in the schools allocated to the control group. Of their 5406 household contacts, 3184 (58·90%) were in the households of the influenza vaccine group and 2222 (41·10%) were in the households of the control group, the study sample therefore consisting of 7148 individuals. The majority of household contacts had not received the 2009 seasonal influenza vaccine. The Brazilian influenza vaccine policy in 2009 targeted the population above 60 years of age, and in the vast majority of households included in the study, there were no people in this age group. Only 3·6% of household contacts were in this age group. Of them 41·7% had received the seasonal influenza vaccine in 2009. Their proportional distribution is similar to distribution of the household contacts between the intervention groups.

Of the 1021 children in the influenza vaccine group, 50 were under 9 years of age and 36 (72·0%) received a second dose of the influenza vaccine. Of the 721 children in the control group, 242 were under 9 years of age and 84 (34·7%) received the varicella vaccine. The vaccinated children were in the 6- to 19-year age bracket. The mean age was 11·0 years (95% CI, 10·9–11·1). Of the vaccinated children, 52·6% were female. Age distribution was heterogeneous: of the children in the influenza vaccine group, 85·9% were in the 10- to 14-year age bracket, as were 44·0% of those in the control group. As can be seen in Table1, the two groups were significantly different in terms of age distribution. Among household contacts, those in the 20- to 49-year age bracket predominated, accounting for 56·71% of the sample as a whole.

**Table 1:** Description of the study sample. São Paulo, Brazil, 2009

Characteristic	Schoolchildren					Household contacts				
	Influenza vaccine		Control*		*P*	Influenza vaccine		Control		*P*
	(*n *=* *1021)		(*n *=* *721)**			(*n *=* *3148)		(*n *=* *2222)**		
	*n*	%	*n*	%		*n*	%	*n*	%	
Gender										
Female	529	51·8	388	53·8	0·41	1690	53·1	1214	55·1	0·001
Male	492	48·2	333	46·2		1494	46·9	989	44·9	
Age bracket										
0–9	955	93·5	696	96·5	0·001	512	16·6	363	17·1	0·002
10–19	66	6·5	25	3·5		503	16·3	376	17·7	
20–49	–	–	–	–		1728	56·2	1223	57·5	
≥50	–	–	–	–		334	10·9	165	7·8	

* Control Group: Meningococcal C conjugate vaccine and varicella vaccine.

** Control Group: Totals are slight different due to missing values.

Regarding self-reported race, 46·4% of the schoolchildren described themselves as White, whereas 45·5% described themselves as mixed race. The remaining defined themselves as Black (6·7%), Asians (0·7%), and Indigenous (0·7%).

Regarding the level of education, 32·2% of the mothers or legal guardians had finished high school. A total of 40·0% of the vaccinated schoolchildren lived in households with three rooms, and 34·4% of the households had four residents. In 35·5% of the households, the schoolchild shared a room with another resident.

### Adverse events

Seventy (4·0%) children reported adverse events temporally associated with vaccination in our study. The majority of them were mild reactions at the injection site. Only one of them was classified as severe and required medical attention. It was a case of cellulitis at the injection site. The schoolchild in question, allocated to the experimental group, received treatment and advice from the physician on our research team and recovered without sequelae.

### Outcomes

A total of 632 cases of ARI (as defined in this study) were detected. Of those, 355 (56·2%) occurred in the influenza vaccine group and 277 (43·8%) occurred in the control group. Since the beginning of active surveillance (epidemiological week 22), there had been circulating agents causing respiratory infections. However, the peak occurrence of ARI, LCI, seasonal influenza virus infection, and influenza A(H1N1)pdm09 virus infection was between epidemiological weeks 26 and 28 for both groups.

Of the 632 cases of ARI, 204 occurred among the schoolchildren. Of those 204 ARI cases, 115 occurred in the influenza vaccine group and 89 occurred in the control group. The incidence density of ARI was 3·71 cases per 1000 person/weeks, being 3·53 per 1000 person/weeks in the influenza vaccine group and 3·97 per 1000 person/weeks in the control group. Thirty-five subjects presented ARI more than once during the active surveillance period; however, in the analysis, the repeated episodes were not considered. Of the 632 cases of ARI, 103 (16·3%) were classified as cases of LCI after specific laboratory tests. Of those 103 LCI cases, 55 (53·4%) were confirmed as cases of influenza A(H1N1)pdm09 virus infection and 48 (46·6%) were confirmed as cases of seasonal influenza viruses infection. Genotypic characterization of the 48 cases of seasonal influenza virus infection confirmed that 25 (52·1%) were cases of influenza A(H3) virus infection and 1 was a case of influenza B virus infection. It was impossible to characterize the remaining 23 cases, once there were insufficient nasal secretion samples available to perform the analysis.

Table2 shows that the incidence density of ARI, LCI, seasonal influenza viruses infection, and influenza A(H1N1)pdm09 virus infection was higher in the control group than in the influenza vaccine group (3·97, 0·72, 0·34, and 0·36 cases per 1000 person/weeks, respectively, in the former group), being LCI and seasonal influenza significant different. In addition, the rates for ARI, LCI, seasonal influenza viruses infection, and influenza A(H1N1)pdm09 virus infection were higher in the schoolchildren than in the household contacts (5·37, 1·30, 0·54, and 0·66 cases per 1000 person/weeks, respectively, in the former group).

**Table 2:** Incidence density of the study outcomes (acute respiratory infection, laboratory-confirmed influenza, seasonal influenza viruses infection, and influenza A[H1N1]pdm09 virus infection), with respective adjusted incidence rate ratios and their 95% CI. São Paulo, Brazil, 2009

	Flu vaccine		Control group		95% confidence interval		
	Cases	ID*	Cases	ID*	IRR	Lower limit	Upper limit
School children							
ARI	115	4·85	89	5·37	0·90	0·75	1·08
LCI	27	1·06	23	1·30	0·82	0·30	0·89
Seasonal influenza	13	0·50	10	0·54	0·93	0·16	0·79
Influenza A (H1N1)	15	0·58	12	0·66	0·88	0·29	1·28
HC							
ARI	240	3·12	188	3·53	0·88	0·74	1·07
LCI	22	0·27	31	0·55	0·49	0·29	0·86
Seasonal influenza	9	0·11	16	0·28	0·39	0·16	0·80
Influenza A (H1N1)	13	0·16	15	0·26	0·62	0·29	1·27
All							
ARI	355	3·53	277	3·97	0·89	0·79	1·07
LCI	49	0·47	54	0·72	0·65	0·40	0·88
Seasonal influenza	22	0·20	26	0·34	0·59	0·29	0·91
Influenza A (H1N1)	28	0·26	27	0·36	0·72	0·39	1·13

ID, incidence density; IRR, incidence rate ratios; ARI, acute respiratory infection; HC, household contact; and LCI, laboratory-confirmed.

* Per 1000 person/weeks.

### Effectiveness of vaccination

As can be seen in Table3, influenza vaccination was effective in preventing influenza, being most effective in preventing seasonal influenza and LCI among the schoolchildren, as well as among their in household contacts, and in the sample as a whole. No difference in vaccine effectiveness was observed for the children 8 years of age and younger that received one or two doses of the influenza vaccine. Regarding the remaining outcomes, their 95% CI included the null value.

**Table 3:** Protective effectiveness of influenza vaccination for the study outcomes [acute respiratory infection, laboratory-confirmed influenza, seasonal influenza virus infection, and influenza A(H1N1)pdm09 virus infection] among the schoolchildren and their household contacts. São Paulo, Brazil, 2009

		95% Confidence Interval	
Category	Effectiveness (%)	Lower limit	Upper limit
School children			
ARI	11·0	−8·0	25·0
LCI	48·0	11·4	69·7
Seasonal influenza	65·0	20·9	84·5
Influenza A (H1N1)	39·0	−27·8	71·1
HC			
ARI	11·0	−7·2	25·9
LCI	50·0	14·1	71·1
Seasonal influenza	65·0	19·6	84·3
Influenza A (H1N1)	40·0	−27·0	71·2
All			
ARI	8·0	−6·9	21·3
LCI	40·0	12·2	59·5
Seasonal influenza	49·0	9·4	71·0
Influenza A (H1N1)	34·0	−13·2	61·1

LCI, laboratory-confirmed influenza; ARI, acute respiratory infection; and HC, household contacts.

## Discussion

This was the first community trial in Brazil in which the influenza vaccine was used as an experimental intervention. The strategy of vaccinating children in schools proved to be simple and feasible. However, the incidence density for LCI was much lower than expected, although active surveillance allowed us to detect the majority, if not all symptomatic cases in the study population (all households in the sample having been contacted at least on a weekly basis). The expected incidence (between 2 and 4%) was estimated considering unpublished data from tropical settings in Asia. Even there, the observed incidence was lower than the preliminary reports. 18

In the present study, we described the occurrence of ARI, seasonal influenza virus infection, and influenza A(H1N1)pdm09 virus infection in a community sample. Vaccination of children and adolescents with the 2009 Southern Hemisphere trivalent inactivated seasonal influenza vaccine conferred indirect protection against seasonal influenza and LCI in unvaccinated household contacts. This result is consistent with those of other randomized clinical trials, in which influenza vaccination was found to provide significant protection against variously defined respiratory diseases in household contacts,^5^,^6^,^8^,^19^ vaccine effectiveness having been found to be 16–30% for respiratory tract infections 1 and 24–42% for respiratory infections with fever.^5^,^19^ It is important to emphasize that the 2009 Southern Hemisphere influenza vaccine composition did not include the pandemic strain, which emerged after the vaccine had been manufactured. Despite this limitation, the present trial detected a number of positive samples for influenza viruses other than the pandemic one.

A very small proportion of vaccinated children reported adverse events temporally associated with vaccination. This finding might be related to high importance attributed to vaccination in Brazilian society. Mild adverse events tend to be disregarded by parents. Mild adverse events tend not to be reported by parents. 20

One of the limitations of the present study was a delay in obtaining permission to begin the preparations for vaccination in the selected schools. Because of the delay, our schedule of meetings of parents could not be completed. So, a high proportion of parents or legal guardians did not have the opportunity to ask questions regarding vaccination and therefore did not allow their children to participate in the study. Of all eligible children, 66·5% did not participate in the study, the final sample size being therefore smaller than estimated. This reduced the power of the study. The extent of this problem varied across schools. This resulted in an imbalance among vaccination rates (which varied widely across schools) and also of age distribution. In addition, it is possible that cases of ARI/influenza occurring early in the influenza season (before vaccination and active surveillance) went undetected because of the delay in vaccinating the children.

Another limitation of the present study was a lack of resources for genotypic characterization of the samples collected from participants. This precluded the description of the profiles of circulating strains and the comparison of those strains with the influenza vaccine strains. In addition, the lack of resources precluded the identification of other etiologic agents in nasal secretion samples testing negative for influenza. In 529 (83·70%) of the 632 cases of ARI, the samples remained undiagnosed. Of those 529 samples, 223 (42·16%) were from the control group and 306 (57·84%) were from the influenza vaccine group.

Finally, the 2009 influenza A (H1N1) pandemic occurred concomitantly with seasonal influenza. This unexpected event certainly affected the results regarding the incidence of influenza and the effectiveness of influenza vaccination, given that the concordance between the influenza vaccine strains and the circulating strains was very low. The emergence of the pandemic strain obliged us to include it as one of the study outcomes.

However, in spite of the limitations, the present study confirmed the direct and indirect effectiveness of influenza vaccination in protecting vaccinated schoolchildren and their unvaccinated household contacts. Because of the high morbidity rates among schoolchildren and the role that schoolchildren play in disease transmission and maintenance, vaccination of such children can be a very interesting strategy to be adopted in Brazil as a way of increasing vaccination coverage and allowing a greater number of individuals to remain disease free.^11^ Given that some countries have implemented influenza vaccination programs for school-age children,^21^,^22^ the results of the present study can guide further studies examining this issue as a way of providing public health policymakers with a basis for improving and expanding the vaccination strategy currently used in Brazil, to reduce the burden of influenza in the country. In years the following the pandemic, the Brazilian influenza vaccination policy has been changed. The yearly vaccine campaigns now include pregnant women and children below 5 years of age. More studies are still needed to clarify the direct and indirect effects of the influenza vaccination of schoolchildren in tropical settings such as Brazil.

## Footnotes

* Dr. P. Kilgore, personal communication.
